# Expression of A disintegrin and metalloprotease 8 is associated with cell growth and poor survival in colorectal cancer

**DOI:** 10.1186/1471-2407-14-568

**Published:** 2014-08-07

**Authors:** Zuli Yang, Yang Bai, Lijun Huo, Hao Chen, Jintuan Huang, Jizheng Li, Xinjuan Fan, Zihuan Yang, Lei Wang, Jianping Wang

**Affiliations:** Department of Gastrointestinal Surgery, The Sixth Affiliated Hospital of Sun Yat-sen University (Guangdong Gastrointestinal and Anal Hospital), Sun Yat-Sen University Guangzhou, Guangzhou, P.R. China; Department of Ophthalmology, The First Affiliated Hospital of Sun Yat-sen University, Sun Yat-Sen University Guangzhou, Guangzhou, P.R. China; Gastrointestinal Institute, Sun Yat-Sen University Guangzhou, Guangzhou, P.R. China; Department of Colon & Rectum Surgery, The Sixth Affiliated Hospital of Sun Yat-sen University (Guangdong Gastrointestinal and Anal Hospital), Sun Yat-Sen University Guangzhou, 26 YuancunErheng Road, Guangzhou, 510655 P.R China

**Keywords:** Colorectal cancer, A disintegrin and metalloprotease 8 (ADAM8), Proliferation, Prognosis, Overall survival, Disease free survival

## Abstract

**Background:**

A disintegrin and metalloprotease 8 (ADAM8) has been reported to be associated with various malignancies. However, no studies have examined ADAM8 association in colorectal cancer (CRC). The aim of this study was to investigate the expression and function of ADAM8 in CRC.

**Methods:**

Expression level of ADAM8 in CRC was evaluated by quantitative RT-PCR, western blot and immunohistochemical staining analysis. The role of ADAM8 in colorectal carcinogenesis was evaluated by *in vitro* assays. The correlations between ADAM8 status and clinicopathological features including survival were analyzed.

**Results:**

ADAM8 was highly expressed in CRC tissues compared with adjacent normal tissues. Knockdown of ADAM8 in two CRC cell lines resulted in reduced cellular growth and proliferation, and increased apoptosis. Immunohistochemistry analysis showed no significant correlations of ADAM8 protein expression with clinicopathologic features. Survival analysis indicated that patients with ADAM8-positive tumors had worse 5-year overall survival (OS, *p* = 0.037) and 5-year disease free survival (DFS, *p* = 0.014) compared with those with ADAM8-negative tumors. Multivariate analysis indicated ADAM8 expression was an independent prognostic factor for both OS and DFS (both *p*< 0.001). Subgroup analysis showed that 5-year OS of colon cancer, T3-T4 stage and N0 stage was worse for patients with ADAM8-positive tumors than those with ADAM8-negative tumors (*p*< 0.05). The 5-year DFS in colon cancer, T3-T4 stage, N0 stage, TNM stage II, adenocarcinoma, moderate differentiation and male patient subgroups was also worse for patients with ADAM8-positive tumors than those with ADAM8-negative tumors (*p* < 0.05).

**Conclusions:**

Our results show that ADAM8 is overexpressed in CRC, promotes cell growth and correlates with worse OS and DFS, and thus could serve as a biomarker for individual CRC patient therapy.

## Background

Colorectal cancer (CRC) is the third most common cancer and the fifth leading cause of cancer-related deaths, with approximately 715,000 new cases and 70,000 deaths annually in China. The survival of CRC patients is closely correlated with conventional and clinicopathological characteristics, such as tumor location, differentiation grade and TNM stages [1]. However, in practice, CRC patients with the same pathological features may have different prognosis. To discover new treatment options and more precise assessment of this malignancy, some potential therapy targets and candidate biomarkers have been reported, such as adenomatous polyposis coli (APC) gene, K-RAS gene, p53 gene and microsatellite instability (MSI) [2]. Among these targets and candidate biomarkers, some are used to justify whether adjuvant therapy is suitable for individual CRC patients, including MSI and wild-type or mutation of K-RAS and BRAF in certain exons [3–5].

A disintegrin and metalloprotease 8 (ADAM8) is a member of the human ADAM family, which contains disintegrin and metalloprotease domains [6]. ADAM proteins are involved in cell adhesion, cell migration, cell fusion, membrane protein shedding and proteolysis [7, 8]. Aberrant expression of ADAM8 has been identified in solid tumors, such as gliomas, lung cancer [9], pancreatic cancer [10], renal cell carcinomas [11] and prostate carcinomas [12]. ADAM8 overexpression has been associated with poor prognosis in hepatocellular carcinoma [13], breast cancer [14] and pancreatic adenocarcinoma [10], and might act as a potential therapeutic target. Mechanistically, ADAM8 is involved in tumorigenesis by stimulating angiogenesis [14, 15], increasing cellular abilities of invasion and migration [10, 14], and inhibiting cancer cell apoptosis [16]. Previous studies showed that ADAM9 [17], ADAM10 [18], ADAM17 [19], ADAM23 [20] and ADAM29 [21] were involved in colorectal tumorigenesis and that ADAM8 was involved in lymph node metastasis of gastric cancer. However, the possible role of ADAM8 in CRC has not yet been evaluated. In the present study, we report the identification of ADAM8 as a novel biomarker and a potential prognostic indicator, and also provide evidence for its possible role in human colorectal carcinogenesis.

## Methods

### Tissue samples, cell culture and cDNA preparation

Thirty CRC tissue samples sets (each containing tumor and adjacent tissues) were obtained from the Sixth Affiliated Hospital of Sun Yat-sen University. Adjacent normal tissues were obtained at a distance of more than 5 cm from the tumor margin and confirmed by a pathologist. Eight human colorectal adenocarcinoma cell lines (HCT8, HT29, SW620, SW480, DLD1, HCT116, LOVO and CACO2) were purchased from the Culture Collection of Chinese Academy of Science (Shanghai, China), and cultured in RPMI 1640 supplemented with 10% fetal bovine serum (Hyclone, USA) and 1% penicillin-streptomycin at 37°C in a 5% CO_2_ incubator.

Total RNA from human CRC tissues cells was prepared using Trizol reagent (Invitrogen, Carlsbad, CA). Reverse transcription was performed using the ReverTra Ace qPCR RT Kit (TOYOBO CO., Osaka, Japan) according to the manufacturer’s instructions.

This study was approved by the institutional review boards of Sun Yat-Sen University (Guangzhou, China), and written informed consent was obtained from each patient in this study.

### Tissue microarray (TMA)

Three hundred and forty-two CRC samples were obtained from the tumor bank of the Department of Pathology of Sun Yat-Sen University (Guangzhou, China). The patients underwent initial surgical resection for CRC between January 2000 and November 2006 and were followed up until April 2010 to collect general information, pathology reports, and information regarding the patients’ conditions after surgery. The samples were formalin-fixed and paraffin-embedded.

TMAs were constructed using an automated TMA instrument (ALPHELYS, Plaisir, France). After identifying the hematoxylin and eosin (H&E)-stained slides for optimal tumor tissue, two cylindrical core biopsies (1 mm diameter) were punched from each formalin-fixed, paraffin-embedded tissue block and arrayed in recipient TMA blocks (2 × 3 cm) as previously described [22].

### RNA interference (RNAi)

ADAM8 siRNA oligonucleotides (si-ADAM8-1 sense 5’-GGACAAGCUAUAUCAGAAAdTdT-3’ and antisense 3’-dTdTCCUGUUCGAUAUAGUCUUU-5’; and si-ADAM8-2 sense 5’-GCACCUGCAUGACAACGUAdTdT-3’ and antisense 3’-dTdTCGUGGACGUACUGUUGCAU-5’) and siRNA control oligonucleotides were obtained from RiboBio Co. Ltd (Guangzhou, China). HT29 and SW480 cells (1 × 10^5^) were cultured in six-well plates until 50% confluence and transfected with 100 nM of the indicated siRNA using LipofectamineImax (Invitrogen, CA, USA) according to the manufacturer’s instructions. The effects of siRNA silencing were analyzed after 48 h transfection. All experiments were repeated three times.

### Quantitative real-time polymerase chain reaction (qRT-PCR)

PCR was performed with each reaction containing 50 ng of reverse-transcribed RNA and 1 μM 5’ and 3’ primers in a 20 μL reaction. The primers used are listed in Table 1. The reaction was performed on an ABI 7500 real-time PCR machine (Applied Biosystems, Foster City, CA, USA) using the following conditions: 95°C for 2 min, 40 cycles of 95°C for 15 sec, and 60°C for 1 min. Briefly, the relative RNA levels in each sample were determined by performing standard curves. β-actin levels were used for normalization.

**Table 1:** **Primers used for qRT-PCR**

Name	Primer sequence forward	Primer sequence reverse
ADAM8	5’-ACAATGCAGAGTTCCAGATGC-3’	5’-GGACCACACGGAAGTTGAGTT-3’
β-actin	5’-CAATGAGCTGCGTGTGGCT-3’	5’-TAGCACAGCCTGGATAGC AA-3’

### Immunohistochemistry (IHC) staining

IHC was performed using the Polink-2 plus^®^ Polymer HRP Detection System (GBI, USA) according to the manufacturer’s instructions. After deparaffinization in xylene and rehydration through a graded alcohol series, slides were transferred to sodium citrate buffer (Beijing DingguoChangsheng Biotech Co. Ltd, AR-0511) for 15 min in the microware and left at room temperature for 30 min. After blocking endogenous peroxidase, slides were incubated with 10 μg/ml goat polyclonal antibody specific to human ADAM8 (R&D Systems, Inc., Minneapolis, MN) at 4°C overnight. Slides were washed three times with phosphate-buffered saline (PBS) and incubated with Polymer Helper (reagent 1, Polink-2 plus^®^ supply) and Poly-HRP anti-Goat IgG (reagent 2, Polink-2 plus^®^ supply) for 30 min. Then the slides were stained with DAB and counterstained with hematoxylin. A negative control using antibody dilution as a substitute for primary antibody was performed for each experiment.

ADAM8 staining was examined by two pathologists blinded to clinicopathological data. Representative fields were captured under low power (100 × magnification) and high power (400 × magnification) by a Leica DMI 4000B inverted microscope (Leica Micro-systems, Wetzlar, Germany). Disagreements were reevaluated until a consensus was reached. IHC staining was analyzed using the Image Pro-Plus (version 6.0, Media Cybernetics, Silver Spring, USA) introduced by Xavier [23]. Briefly, the tumor area was selected as the area of interest (AOI), and the area sum and integrated optical density (IOD) of the AOI were selected as the measurement parameters. ADAM8 expression index equaled the quotient between the IOD and the total area of AOI. The mean expression index for each duplicate was used for statistical analysis. Selection of cutoff value was performed according to a previous study [24]. The cutoff point was 9.79 based on the patient’s OS and DFS reaching significant difference. The CRC tissues were classified based on ADAM8 density into the negative group (less than or equal to 9.79) or positive group (more than 9.79). The ADAM8 positive group in cancer tissues and normal tissues was divided into three subgroups of weak (9.79–64.5), moderate (64.5–111.2) and strong (111.2–256.7) expression according to the IHC scores based on OS and DFD reaching significant difference.

### Western blot

After 72 h transfection, HT29 and SW480 cells were washed three times with PBS and lysed with RIPA buffer (Dingguo, Beijing, China) supplemented with phenylmethanesulfonyl fluoride (PMSF, Dingguo, Beijing, China). ADAM8 protein levels were determined using two-color fluorescent western blotting on the Odyssey infrared imaging system (LI-COR, Nebraska, USA). In brief, protein samples were separated by 10% sodium dodecyl sulfate-polyacrylamide gel electrophoresis (SDS-PAGE) and transferred to a polyvinylidene fluoride (PVDF) membrane (Pall, New York, USA). Membranes were then blocked with 5% skim milk for 1 h. Proteins were detected using mouse monoclonal antibodies specific to human ADAM8 (diluted 1:250, Abcam, UK, ab89127) and β-actin (diluted 1:10,000, Protein Tech, Chicago, USA). After incubating with primary antibodies overnight at 4°C and species-appropriate fluorescently conjugated secondary antibodies for 1 h at room temperature, the blots were observed using the Odyssey infrared imaging system. Secondary antibodies were purchased from Santa Cruz Biotechnology (CA, USA) unless otherwise indicated.

### Cell viability and cell proliferation assay

HT29 and SW480 cells were seeded in 96-well plates at a density of 1 × 10^4^ cells/well. Cells were transfected with ADAM8 siRNA and cell viability was determined 0, 1, 2, 3, and 4 days later using the CellTiter 96 Aqueous One Solution Cell Proliferation Assay kit (Promega, Madison, WI) according to the manufacturer’s protocol. After 72 h of transfection with ADAM8 siRNA, cell proliferation assay was performed using an EDU (5-ethynyl-2’-deoxyuridine) Cell Proliferation Kit (Invitrogen, Camarillo, CA) according to the manufacturer’s instructions. Data are presented as mean ± SD for three independent experiments compared with the control group, and each experiment was performed in triplicate.

### Cell apoptosis assay

HT29 and SW480 cells transfected with ADAM8 siRNA were seeded in 12-well plates at a density of 1 × 10^4^ viable cells/well. After 72 h culture, the cells were fixed in 70% ethanol and stained with 50 mg/ml propidium iodide (BD Pharmingen, San Jose, CA, USA), then sorted by FACSCalibur (BD Biosciences, Franklin Lakes, NJ, USA). Cell cycle profiles were analyzed by ModFit 3.0 software (Verity Software House, Topsham, ME, USA). Apoptosis was determined by dual staining with Annexin V:FITC and propidium iodide (Invitrogen). The Annexin V-positive cells were counted as apoptotic cells.

### Statistical analyses

SPSS 16.0 for Windows (SPSS, Inc., Chicago, IL) was used for statistical analyses. Continuous variables were expressed as mean ± SD and analyzed by t-test. The Chi-square test was used to show differences of categorical variables. Survival analysis was performed using the Kaplan-Meier method and compared using the log-rank test. *P*< 0.05 was considered statistically significant.

## Results

### Expression status of ADAM8 gene in human CRC tissues and cell lines

**Figure 1:**
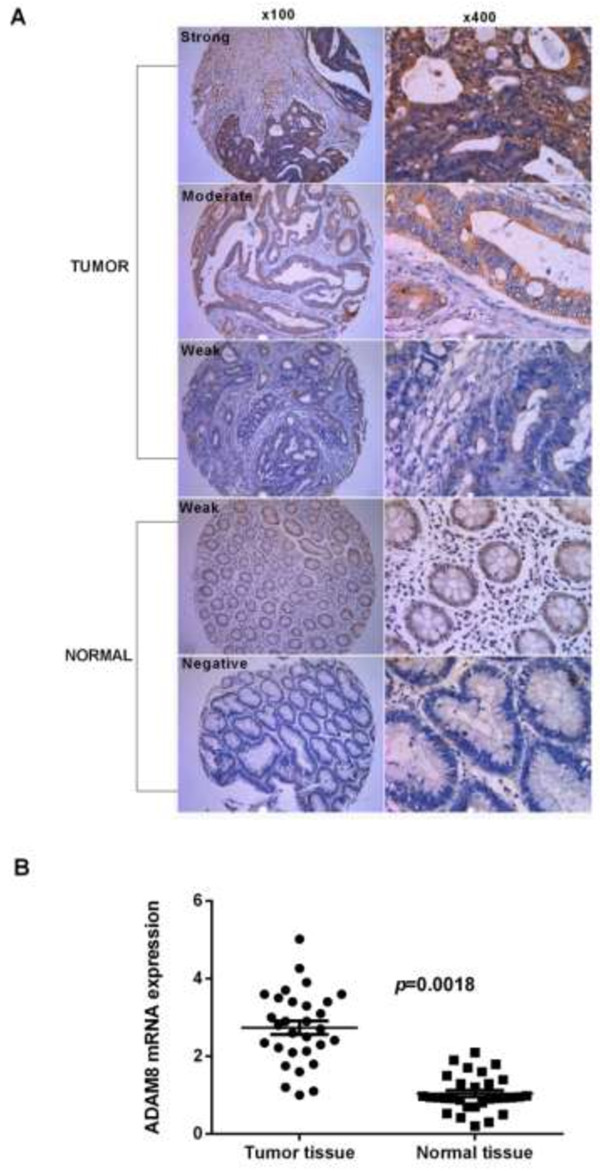
**ADAM8 expression status in CRC tumor and normal tissues. A**, Expression of ADAM8 in tumor and normal colon tissues detected by IHC. Representative images of IHC staining of colon tissues with anti-ADAM8 antibody on tumor and normal tissues (100× for left panel and 400× for right panel). **B**, Mean expression levels of ADAM8 mRNA in tumor tissues were significantly higher than that observed in normal tissues (*p* = 0.0018). The expression of ADAM8 mRNA was normalized to β-actin mRNA, which served as a control for the input cDNA.

We evaluated the expression of ADAM8 protein and mRNA levels in 30 pairs of fresh-frozen CRC tissues and adjacent normal tissues by IHC and qRT-PCR. IHC results showed that specific ADAM8 staining was mainly detected in the cytoplasm and membrane of noncancerous and malignant epithelial cells. IHC staining indicated more CRC tissues with positive ADAM8 expression than in corresponding adjacent normal tissues (81.0% vs. 33.3%, respectively; *p*< 0.0001) (Figure 1A, Table 2). Among the 24 CRC patients with ADAM8 positive tumor tissues, high and moderate expression of ADAM8 was detected in 20 cases and weak expression in 4 cases. Among paired adjacent normal tissues, weak expression of ADAM8 protein was found in 10 cases, while no cases showed high or moderate expression. The mRNA expression levels of ADAM8 were evaluated by qRT-PCR and representative data are shown in Figures 1B and 1C. The mean expression levels of ADAM8 mRNA were significantly higher in tumor tissues compared with those in adjacent normal tissues (2.74 ± 0.17 vs. 1.04 ± 0.09, respectively; *p* = 0.0018). The expression of ADAM8 mRNA was normalized to β-actin mRNA, which served as a control for the input cDNA.

**Table 2:** **Comparison of ADAM8 protein level in normal and tumor tissues by IHC (n = 42)**

Tissue	ADAM8 expression		χ2	***p***value
	Negative	Positive		
Normal	20(66.7%)	10(33.3%)	13.3	<0.0001
Tumor	6(19.0%)	24(81.0%)		

**Figure 2:**
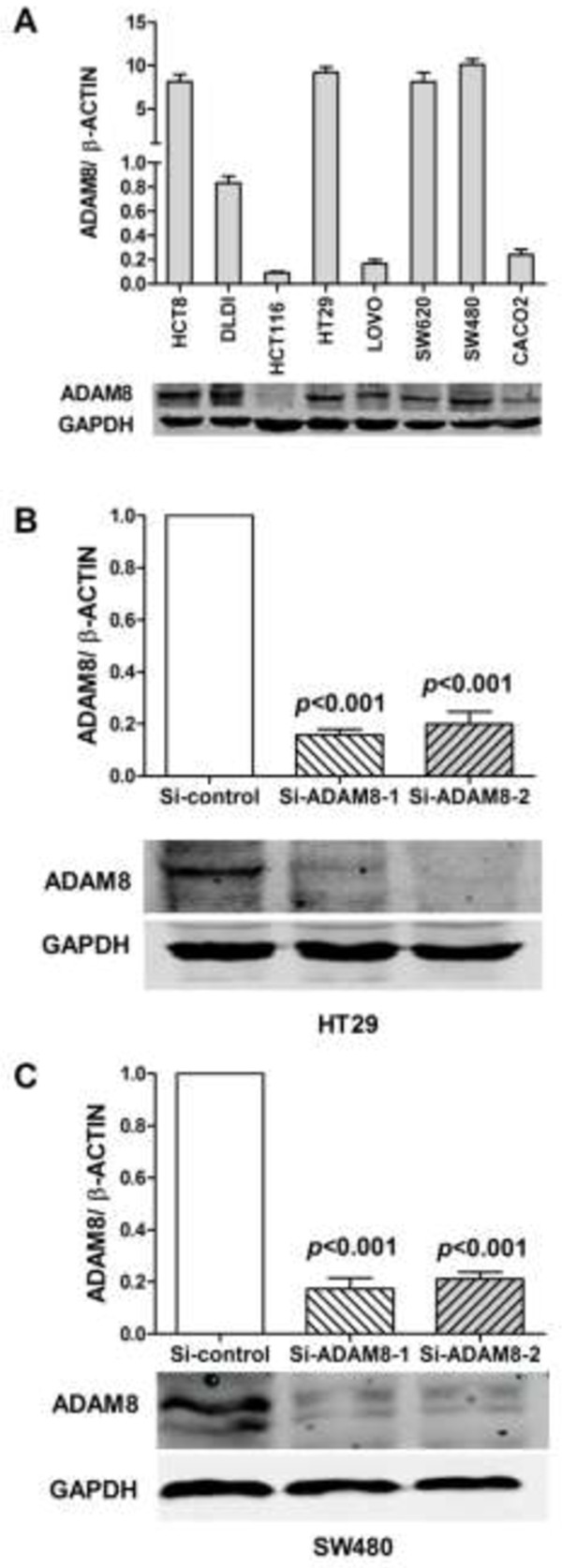
**Expression of ADAM8 in CRC cell lines. A**, Expression of ADAM8 mRNA (top) and protein level (bottom) in HCT8, HT29, SW620, SW480, DLD1, HCT116, LOVO and CACO2 cell lines. **B**, **C**, Significantly decreased expression of ADAM8 mRNA (top) and protein levels (bottom) in ADAM8 siRNA-transfected HT29 cells **(B)** and SW480 cells **(C)** compared with the control group (*p* < 0.001). The expression of ADAM8 mRNA was normalized to β-actin mRNA, which served as the control. Data are expressed as the mean ± standard deviation (SD).

Expression levels of ADAM8 mRNA and protein were also measured in eight CRC cell lines (Figure 2A). The expression level of ADAM8 protein was consistent with mRNA expression level in HCT8, HT29, SW620, SW480, HCT 116 and CACO2 cell lines, but not in DLD1 and LOVO cell lines. Based on these results, we selected HT29 and SW480 cell lines for further analysis. To explore the potential effect of ADAM8 on CRC carcinogenesis, two ADAM8 siRNA oligonucleotides were generated to knockdown ADAM8 expression in HT29 and SW480 cells. Transfection with si-ADAM8-1 decreased ADAM8 mRNA expression levels by 84.3% in HT29 cells (*p* < 0.001) and by 82.7% in SW480 cells (*p* < 0.001) compared with control siRNA. Transfection with si-ADAM8-2 decreased ADAM8 mRNA levels by 82.7% in HT29 cells (*p* < 0.001) and by 78.8% in SW480 cells (*p* < 0.001) (Figure 2B and C, top panel). Western blot analysis confirmed the qRT-PCR results (Figure 2B and C, bottom panel).

### Knockdown of ADAM8 influences proliferation and apoptosis of CRC cells

**Figure 3:**
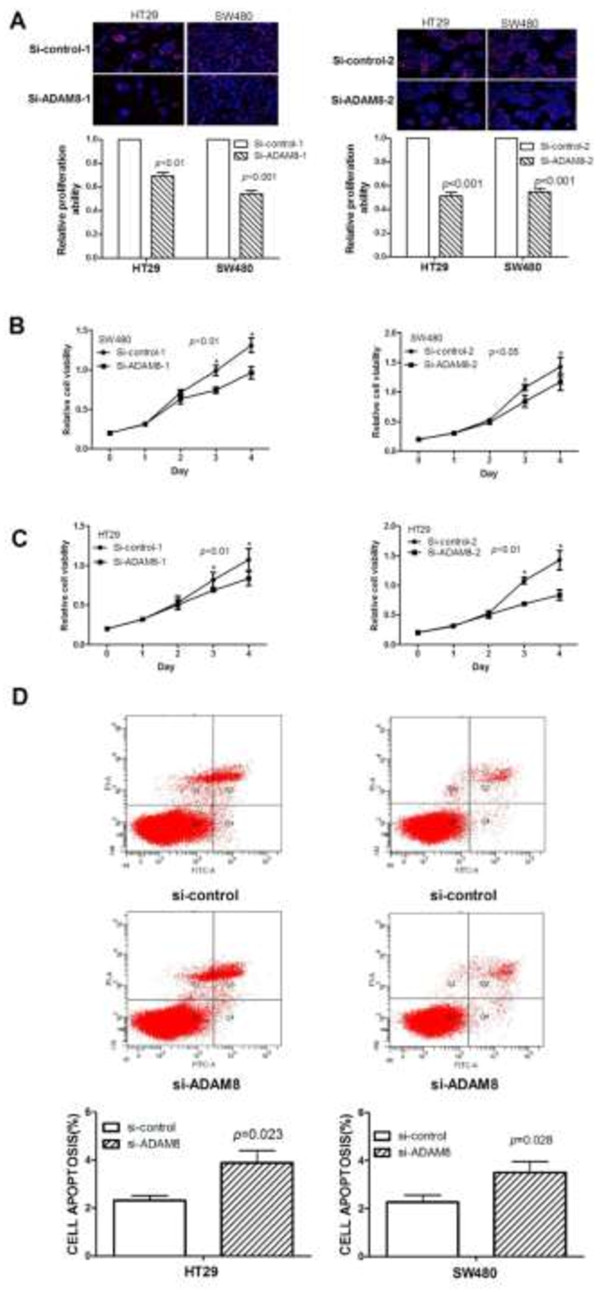
**ADAM8 influences proliferation and apoptosis of CRC cells.** ADAM8 siRNA-mediated reduction of ADAM8 significantly inhibited proliferation **(A)** and growth **(B** **and C)** of HT29 and SW480 cells compared with the control group. **D**, Knockdown of ADAM8 significantly induced cell apoptosis in HT29 and SW480 cells compared with controls.

Cell proliferation assays revealed that si-ADAM8-1-mediated decreased expression of ADAM8 significantly inhibited cell proliferation in HT29 cells (down to 30.5%; *p* < 0.01), and si-ADAM8-2 transfection also inhibited proliferation (down to 48.6%; *p* < 0.001) (Figure 3A). Similar results were observed in SW480 cells transfected with si-ADAM8-1 (down to 45.6%; *p* < 0.001) and si-ADAM8-2 (down to 45.2%; *p* < 0.001) (Figure 3A). Cell viability assay demonstrated that knockdown of ADAM8 significantly inhibited cell growth in both SW480 (Figure 3B) and HT29 cells (Figure 3C). Cell cycle and apoptosis assay showed that the percentage of cell apoptosis in siADAM8-transfected HT29 and SW480 cells was significantly higher than that in control cells (Figure 3D). Together these results suggest that ADAM8 is involved in CRC carcinogenesis by accelerating proliferation/growth and inducing apoptosis of CRC cells.

### Correlation of ADAM8 expression with clinicopathological characteristics and long-term survival of CRC

**Figure 4:**
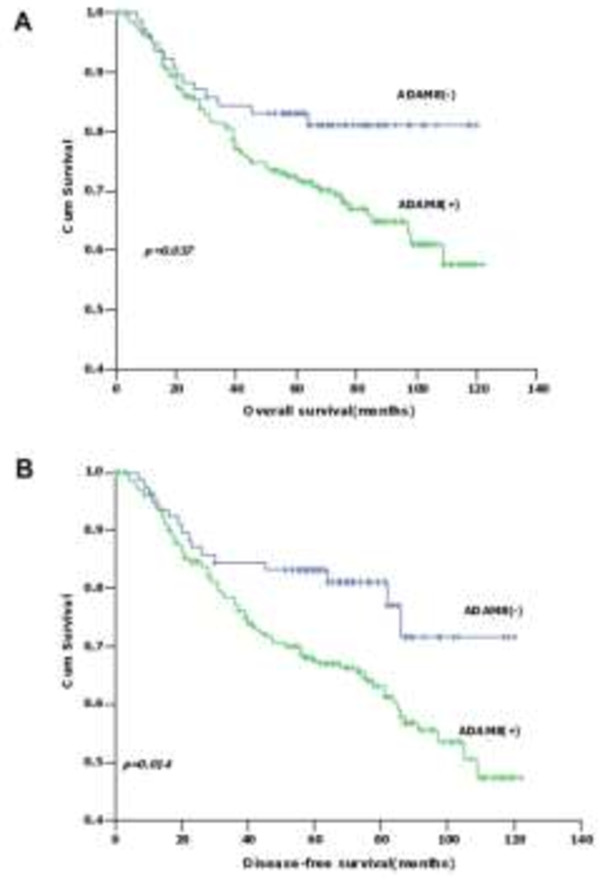
**Survival of CRC patients according to the expression status of ADAM8 protein. A**, CRC patients with positive ADAM8 had poorer OS than those with negative ADAM8 (*p* = 0.037). **B**, CRC patients with positive ADAM8 had poorer DFS than those with negative ADAM8 (*p* = 0.014).

The association between ADAM8 expression and clinicopathological characteristics was assessed in 342 primary CRC patients. IHC was used to detect ADAM8 protein expression status, and tissues were scored as positive or negative as described in Materials and Methods. Among 342 CRC patients, ADAM8 was positive in 261 cases (76.3%) and negative in 81 cases (23.7%). However, no significant correlations were found between ADAM8 expression status and clinicopathological indicators (Table 3). The correlation of ADAM8 protein expression and postoperative survival was also evaluated. Five-year overall survival (OS) and disease-free survival (DFS) for all patients were 73% and 70%, respectively. The 5-year OS for patients with ADAM8 positive tumors was significantly poorer than those with ADAM8 negative tumors (70% vs. 81%, respectively; *p* = 0.037) (Figure 4A). Similar results were found for the 5-year DFS of CRC patients with positive and negative ADAM8 tumors (53% vs. 80%, respectively; *p* = 0.014) (Figure 4B).

**Table 3:** **Association between ADAM8 expression in CRC and clinicopathologic characteristics (n = 342)**

Indicator	ADAM8 expression		χ2	***p***value
	Negative (n = 81)	Positive (n = 261)		
**Gender**			1.448	0.229
Male	49(60.5%)	138(52.9%)		
Female	32(39.5%)	123(47.1%)		
**Age**			0.769	0.380
≤65 yrs	51(63.0%)	150(57.5%)		
>65 yrs	30(37.0%)	111(42.5%)		
**Location**			0.646	0.421
Colon	42(52.9%)	122(46.7%)		
Rectum	39(48.1%)	139(53.3%)		
**T stage**			0.465	0.495
T1-T2	16(19.8%)	43(16.5%)		
T3- T4	65(80.2%)	218(83.5%)		
**N stage**			1.675	0.196
N0	54(66.7%)	153(58.7%)		
N1- N2	27(33.3%)	108(41.3%)		
**M stage**			0.256	0.613
M0	68(84.0%)	225(86.2%)		
M1	13(16.0%)	36(13.8%)		
**TNM stage**			0.544	0.909
I	8(9.9%)	33(12.6%)		
II	33(40.7%)	107(41.0%)		
III	28(34.6%)	83(31.8%)		
IV	12(14.8%)	38(14.6%)		
**Differentiation grade**			2.863	0.239
Well	5(12.3%)	25(7.8%)		
Moderately	71(75.4%)	207(83.0%)		
Poorly	5(12.3%)	29(9.2%)		
**Histological type**			1.209	0.272
Adenocarcinoma	72(88.9%)	219(83.9%)		
Mucinous/Signet-ring adenocarcinoma	9(11.1%)	42(16.1%)		
**CA19-9 (ug/L)**			3.582	0.058
≤60	67(82.2%)	236(90.4%)		
>60	14(17.8%)	25(9.6%)		
**CEA (ug/L)**			0.225	0.635
≤5	52(64.2%)	175(67.0%)		
>5	29(35.8%)	86(33.0%)

**Figure 5:**
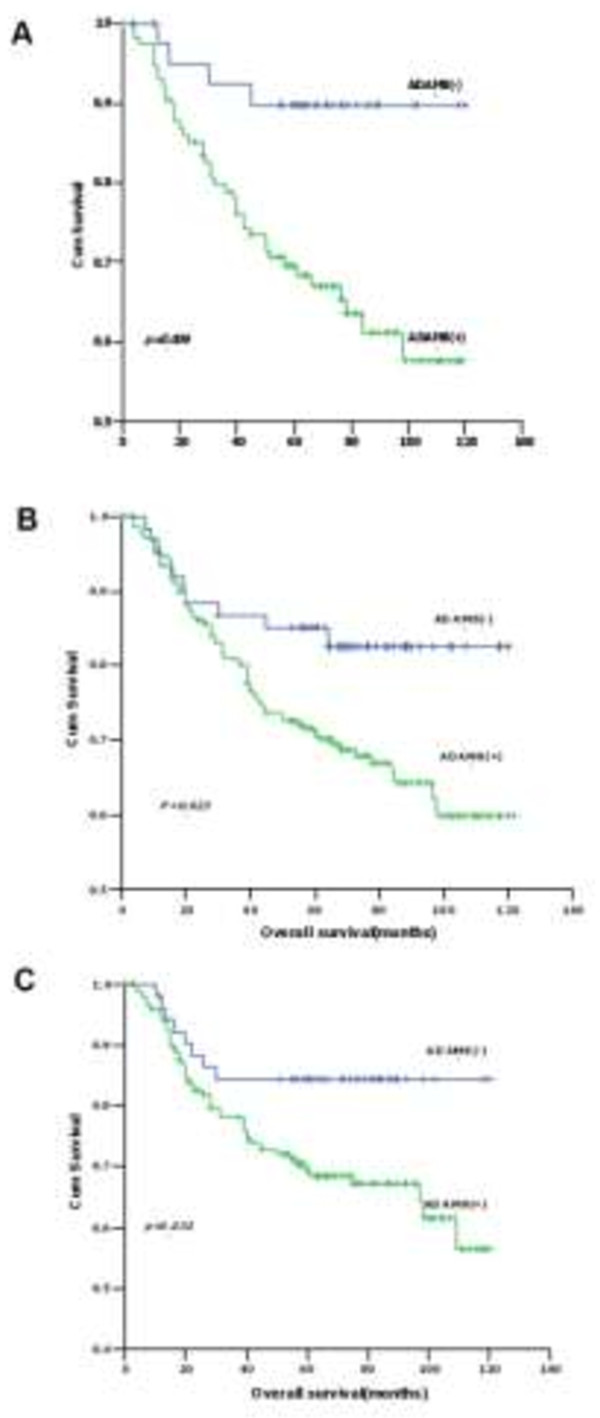
**Five-year OS of patients with positive ADAM8 versus negative ADAM8 tumors in (A) colon cancer (** ***p*** **= 0.006), (B) T3/4 depth of invasion (** ***p*** **= 0.023) and (C) N0 stage (** ***p*** **= 0.032).**

Regarding 5-year OS, univariate analysis indicated that T stage, N stage, M stage, TNM stage, differentiation grade, preoperative CEA and CA19-9 levels, and ADAM8 protein expression status were found to be prognostic factors (Table 4). However, multivariate analysis demonstrated that TNM stage, preoperative CEA levels and ADAM8 protein expression status (HR = 1.943; 95% CI: 1.089–3.465, *p* = 0.024) were independent prognostic indicators (Table 4). Further analysis for OS in each subgroup showed that patients with ADAM8 positive tumors have poorer 5-year OS than those with negative ADAM8 in colon cancer (*p* = 0.006) (Figure 5A), T3/T4 stage (*p* = 0.023) (Figure 5B) and N0 stage (*p* = 0.032) (Figure 5C) subgroups compared with rectal cancer, T1/T2 stage and N1-2 stage patients. No significant correlation was found with other subgroups.

**Table 4:** **Univariate and multivariate analyses of the prognostic factors for 5-year OS of CRC patients (n = 342)**

Indicator	Univariate analysis		Multivariate analysis		
	5-year OS	***p***value	HR	95% CI	***p***value
**T stage**					
T1-T2	79%	0.009			NS
T3-T4	64%				
**N stage**					
N0	78%	<0.0001			NS
N1-N2	61%				
**M stage**					
M0	73%	<0.0001			NS
M1	23%				
**Differentiation grade**					
Well	74%	0.004			NS
Moderately	72%				
Poorly	47%				
**TNM stage**					
I	86%	<0.0001	1	Reference	<0.0001
II	78%		1.016	0.411-2.511	
III	69%		1.674	0.686-4.083	
IV	22%		9.685	3.773-24.862	
**CEA(ug/L)**					
≤5	80%	<0.0001	1	Reference	0.006
>5	54%		1.144	0.465-2.811	
**CA19-9 (ug/L)**		0.040			
≤60	72%				NS
>60	59%				
**ADAM8 protein**					
Negative	81%	0.037	1	Reference	0.024
Positive	67%		1.943	1.089-3.465	

CRC, colorectal cancer; HR, hazard ratio; CI, confidence interval; NS, not significant.

**Figure 6:**
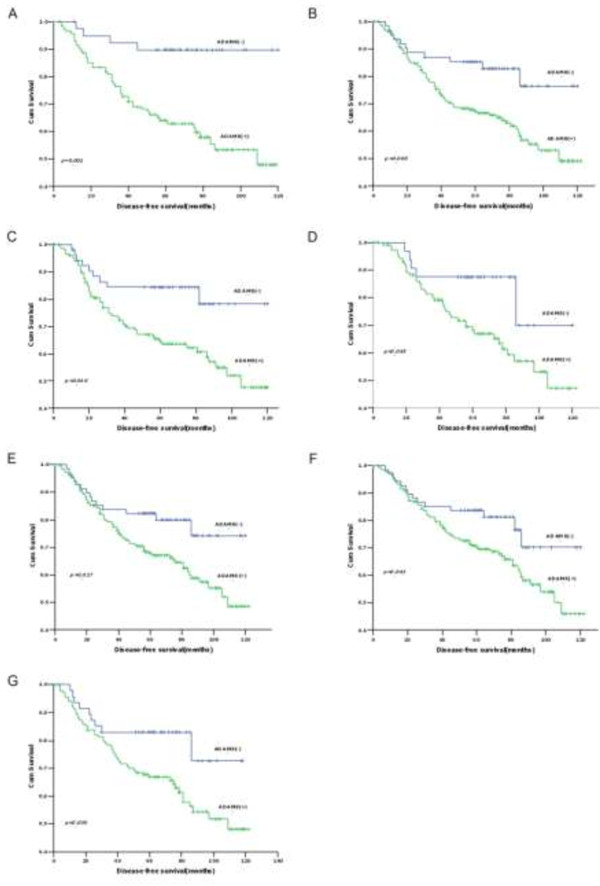
**Five-year DFS of patients with positive ADAM8 versus negative ADAM8 tumors in (A) colon cancer (** ***p*** **= 0.001), (B) T3/4 stage (** ***p*** **= 0.009), (C) N0 stage (** ***p*** **= 0.032), (D) TNM II stage (** ***p*** **= 0.045), (E) adenocarcinoma (** ***p*** **= 0.027), (F) moderate differentiation (** ***p*** **= 0.043) and (G) male CRC patients (** ***p*** **= 0.030).**

Regarding 5-year DFS, N stage, differentiation grade, TNM stage, preoperative CEA and CA19-9 levels, and ADAM8 protein expression status were found to be prognostic factors by univariate analysis (Table 5). However, TNM stage, preoperative CEA levels and ADAM8 protein expression status (HR = 2.108; 95% CI: 1.922–3.480, *p* = 0.025) were independent prognostic indicators by multivariate analysis (Table 5). Further analysis for DFS in subgroups showed that patients with positive ADAM8 expression had poorer 5-year DFS than those with negative ADAM8 expression in colon cancer (*p* = 0.001) (Figure 6A), T3/T4 stage (*p* = 0.009) (Figure 6B), N0 stage (*p* = 0.010) (Figure 6C), TNM II stage (*p* = 0.045) (Figure 6D), adenocarcinoma (*p* = 0.027) (Figure 6E), moderate differentiation (*p* = 0.043) (Figure 6F) and male CRC patients (*p* = 0.030) (Figure 6G) subgroups. No significant correlations were found with other indicators.

**Table 5:** **Univariate and multivariate analyses of the prognostic factors for 5-year DFS of CRC patients (n = 292)**

Indicator	Univariate analysis		Multivariate analysis		
	5-year DFS	***p***value	HR	95% CI	***p***value
**N stage**					
N0	68%	0.031			NS
N1-N2	59%				
**Grade**					
Well	71%	0.003			NS
Moderately	57%				
Poorly	46%				
**TNM stage**					
I	85%	0.017	1	Reference	0.027
II	72%		2.445	0.958-6.236	
III	61%		2.482	0.954-6.457	
**CEA(ug/L)**					
≤5	75%	<0.0001	1	Reference	0.001
>5	52%		2.052	1.342-3.145	
**CA19-9 (ug/L)**					
≤60	69%	0.016			NS
>60	55%				
**ADAM8 protein**					
Negative	81%	0.014	1	Reference	0.025
Positive	67%		2.108	1.922-3.480	

CRC, colorectal cancer; HR, hazard ratio; CI, confidence interval; NS, not significant.

## Discussion

A member of the ADAM family, ADAM8 has been detected in many cell types and various types of cancer [12, 25–30]. However, no study of ADAM8 expression in CRC patients has been performed. In the present study, expression of both protein and mRNA levels of ADAM8 in 30 CRC patients were significantly higher in cancerous tissues than corresponding adjacent normal tissues, suggesting its importance in CRC carcinogenesis. IHC analysis of 342 CRC patients identified 261 (76.3%) cases with positive ADAM8 expression and 81 (23.7%) with negative ADAM8 expression, indicating that ADAM8 is upregulated in human CRC. To explore the potential role of ADAM8 in CRC carcinogenesis, cell proliferation and apoptosis assay were used to assess the influence of ADAM8 on cell growth. Our findings showed that siRNA-mediated downregulation of ADAM8 in CRC cells significantly suppressed cell proliferation and induced cell apoptosis, which is in agreement with previous reports [13, 16]. These data strongly suggest that ADAM8 is involved in CRC carcinogenesis and regulates cell growth by accelerating cell proliferation and inhibiting cell apoptosis. Although previous studies have shown that ADAM8 increases invasion and migration abilities of tumor cells [14, 15, 21], we did not find a significant decrease of invasion and migration in ADAM8 siRNA-transfected cells compared with control cells (data not shown).

In the present study, we explored the relationship between ADAM8 expression status and clinicopathological features in CRC. Although previous studies reported that ADAM8 expression correlates significantly with tumor size, histological differentiation, regional and distant metastasis, tumor stages in several cancers progression [12, 13, 29, 31], we did not find any significant correlations between ADAM8 expression status and any clinicopathological feature in CRC.

In the present study, patients with ADAM8 positive tumors have poorer 5-year OS and DFS than those with ADAM8 negative tumors. Multivariate analysis revealed that ADAM8 positive expression could act as an important factor for unfavorable prognosis in both OS and DFS for CRC patients independent of some conventional indicators, which is in agreement with published papers [25, 29, 30]. Further analysis of survival in patient subgroups suggested that ADAM8 is a prognostic factor for colon cancer but not for rectal cancer, indicating that ADAM8 may not function as a biomarker for rectal cancer. Meanwhile, positive ADAM8 was an adverse indicator for both OS and DFS in T3/T4 depth of invasion and N_0_ stage, and only for DFS in adenocarcinoma, moderately differentiated tumors and male patients. Based on these results, ADAM8 can be considered as a novel prognostic marker for CRC and may serve as a target for individual therapy for certain CRC patients.

Although we explored the expression status, potential roles and clinical implications of ADAM8 in CRC, the underlying mechanism by which ADAM8 influences tumor cell growth and postoperative survival of CRC patients was not investigated in this study. Furthermore, although high expression of ADAM8 induces tumor cell resistance to chemotherapy [16], we were unable to assess the role of post-operative adjuvant chemotherapy with regard to DFS and OS in context of ADAM8 expression in univariate and multivariate analyses due to the shortage of post-operative adjuvant chemotherapy data for 342 CRC patients in this study. More studies investigating these questions should be performed in the future.

## Conclusions

In summary, our results show that ADAM8 is overexpressed in CRC tissues, promoting cancer cell proliferation, inducing cell apoptosis and correlating with worse OS and DFS. Furthermore, ADAM8 may be considered as a novel prognostic marker for CRC and could function as a target of individual therapy for certain CRC patients.

## Abbreviations

ADAM8: A disintegrin and metalloprotease 8
CRC: Colorectal cancer
OS: Overall survival
DFS: Disease-free survival
qRT-PCR: Quantitative RT-PCR.
